# Inhibition of LAMP3 mediates the protective effect of vitamin D against hypoxia/reoxygenation in trophoblast cells

**DOI:** 10.1590/1414-431X2023e12816

**Published:** 2023-10-20

**Authors:** Xiaoyu Tian, Lili Zheng, Jing Ma, Ying Xu, Yulin Zhang, Yalei Pi

**Affiliations:** 1 Department of Pediatrics, The Second Hospital of Hebei Medical University, Shijiazhuang, Hebei, China; 2 Department of Obstetrics, The Second Hospital of Hebei Medical University, Shijiazhuang, Hebei, China

**Keywords:** LAMP3, Vitamin D, Trophoblast, Invasion, Apoptosis, Preeclampsia

## Abstract

Inadequate invasion and excessive apoptosis of trophoblast cells are associated with the development of preeclampsia. Vitamin D deficiency in pregnant women may lead to an increased risk of preeclampsia. However, the underlying mechanisms by which vitamin D is effective in preventing preeclampsia are not fully understood. The objectives of this study were to investigate the role of lysosome-associated membrane glycoprotein 3 (LAMP3) in the pathogenesis of preeclampsia and to evaluate whether vitamin D supplementation would protect against the development of preeclampsia by regulating LAMP3 expression. Firstly, the mRNA and protein levels of LAMP3 were significantly upregulated in the placentas of preeclampsia patients compared to normal placentas, especially in trophoblast cells (a key component of the human placenta). In the hypoxia/reoxygenation (H/R)-exposed HTR-8/Svneo trophoblast cells, LAMP3 expression was also upregulated. H/R exposure repressed cell viability and invasion and increased apoptosis of trophoblast cells. siRNA-mediated knockdown of LAMP3 increased cell viability and invasion and suppressed apoptosis of H/R-exposed trophoblast cells. We further found that 1,25(OH)_2_D_3_ (the hormonally active form of vitamin D) treatment reduced LAMP3 expression in H/R exposed trophoblast cells. In addition, 1,25(OH)_2_D_3_ treatment promoted cell viability and invasion and inhibited apoptosis of H/R-exposed trophoblast cells. Notably, overexpression of LAMP3 abrogated the protective effect of 1,25(OH)_2_D_3_ on H/R-exposed trophoblast cells. Collectively, we demonstrated trophoblast cytoprotection by vitamin D, a process mediated via LAMP3.

## Introduction

Preeclampsia (PE) is a pregnancy disorder characterized by the onset of hypertension and proteinuria after 20 weeks of pregnancy (1). It is the main reason of maternal and fetal morbidity and mortality (2). The regulation of apoptosis is crucial for the survival and differentiation of placenta trophoblasts (3,4). Excessive apoptosis and superficial invasion of trophoblasts result in insufficient spiral artery remodeling, which contributes to the pathogenesis of PE (5,6). Abnormalities in O_2_ concentration, such as persistently low O_2_ concentration (hypoxia) or cycles of hypoxia-reoxygenation, are associated with dysfunction of the placenta in PE (7,8). Therefore, we established a cell model in HTR-8/Svneo trophoblast cells by inducing hypoxia/reoxygenation (H/R) to mimic the pathophysiological characteristics of PE and evaluated the cell biological factor that was dysregulated in PE.

Lysosome-associated membrane glycoprotein 3 (LAMP3) is a member of the lysosome-associated membrane glycoprotein family (9), which participates in the regulation of a variety of cellular processes, such as cell invasion (10), apoptosis (11), and metastasis (12). Previous studies have demonstrated that LAMP3 affects the malignant behavior of many cancers (9-11). The regulatory effect of LAMP3 on the invasive ability and apoptosis of tumor cells has been identified (13,14). A study conducted by Tanaka et al. (11) reported that LAMP3 suppressed epithelial cell growth and contributed to apoptosis. Based on the Gene Expression Omnibus (GEO) dataset (accession number: GSE44711), we found that LAMP3 was upregulated in PE placental samples compared with normal control samples. However, the exact role of LAMP3 in the invasion and survival of trophoblast cells is not well understood.

Vitamin D, through its hormonally active form 1,25-dihydroxyvitamin D3 [1,25(OH)_2_D_3_], exerts essential functions in regulating cell proliferation and differentiation (15,16). A clinical trial has demonstrated that the level of vitamin D is related to the risk of PE (17). Low vitamin D levels in pregnant mice led to symptoms of PE, such as elevated blood pressure and damaged placental development (18). Moreover, vitamin D supplementation could alleviate PE-associated endothelial dysfunction in a rat model (19). Vitamin D facilitated human extravillous trophoblast cell invasion *in vitro* (20). Our previous study found that vitamin D attenuated H/R-induced trophoblast cell injury (21). In the present study, we investigated the molecular mechanisms of vitamin D in preventing preeclampsia.

A previous study demonstrated that LAMP3 expression is downregulated by vitamin D in dendritic cells (22). Nevertheless, it is unclear whether the regulatory relationship in trophoblast cells is consistent with that previously proposed. Therefore, the aim of our study was to determine whether vitamin D inhibits aberrant expression of LAMP3, affects apoptosis and invasion of trophoblast cells, and leads to a protective effect against PE.

## Material and Methods

### Patient samples

Placenta samples were obtained from 23 women: 10 women with PE defined by hypertension (systolic blood pressure ≥140 and/or diastolic blood pressure ≥90 mmHg) and proteinuria (0.3 g/24 h) (23); as a comparative group, 13 normal pregnant women were selected. The clinical characteristics of our study subjects are presented in Table 1. Written informed consent was obtained from all participants. The study was approved by the Research Ethics Committee of the Second Hospital of Hebei Medical University and was carried out in accordance with the Declaration of Helsinki.

**Table 1:** Clinical characteristics of the study subjects.

	Control (n=13)	Preeclampsia (n=10)	P value
Maternal age (years)	30±4.69	31.2±5.095	0.5641
Gestational age (weeks)	37.692±0.947	32.4±3.627	<0.0001
Systolic BP (mmHg)	114.538±5.379	151.2±9.682	<0.0001
Diastolic BP (mmHg)	76.077±6.97	100.2±11.193	<0.0001
Proteinuria (g/24 h)	0.091±0.037	2.919±2.715	0.0011
Neonatal weight (g)	3187.692±338.358	1680±682.837	<0.0001

Data are reported as means±SD. P<0.05 was considered statistically significant (unpaired *t*-test). BP: blood pressure.

### Cell culture

The human villous trophoblast cell line HTR-8/Svneo was obtained from Shanghai Zhong Qiao Xin Zhou Biotechnology Co., Ltd. The cells were maintained in RPMI medium 1640 (Solarbio Science & Technology, Co., Ltd., China) supplemented with 10% fetal bovine serum (Zhejiang Tianhang Biotechnology Co., Ltd., China) at 37°C in a humidified atmosphere with 5% CO_2_.

### Hypoxia/reoxygenation (H/R) exposure

H/R was achieved by exposing HTR-8/Svneo cells to an ambient O_2_ concentration of 2% for 8 h and then for an additional 16 h to an ambient O_2_ concentration of 20%.

### Cell transfection and treatment

Small interfering RNA targeting LAMP3 (siLAMP3), LAMP3 overexpression plasmid (oe-LAMP3), and their corresponding negative controls (siNC and vector) were transfected into HTR-8/Svneo cells using Lipofectamine 3000 (Invitrogen, USA) according to manufacturer's instruction. After transfection for 24 h, cells were cultivated under H/R condition for another 24 h.

For 1,25(OH)_2_D_3_ treatment, H/R-induced HTR-8/Svneo cells were treated with 100 nM 1,25(OH)_2_D_3_ for 24 h.

### Real-time PCR

Total RNA was extracted from placenta cells using TRIpure lysate (BioTeke Corporation, China). RNA was reverse-transcribed with BeyoRT™ II M-MLV reverse transcriptase (Beyotime, China). The reaction was followed by a melting curve protocol according to the specifications of Exicycler^TM^ 96 real-time quantitative thermal block (Bioneer Corporation, Korea). GAPDH was used as an internal control. The primers were as follows: LAMP3 F: CTCGGAGATACTTCAACAT; LAMP3 R: GAGACGGTCAAATAGGC.

### Western blot

Total proteins were extracted using RIPA buffer (Solarbio). Sample proteins were separated by SDS gels and transferred to PVDF membranes (Millipore, USA). The membranes were probed with primary antibodies by LAMP3 antibody (1:500, 12632-1-AP, Proteintech, China), Bcl2 antibody (1:500, A19693, ABclonal, China), Bcl-xL antibody (1:1000, A19703, ABclonal), Bax antibody (1:500, A19684, ABclonal). GAPDH (1:10000, 60004-1-Ig, Proteintech) was used as an internal control. Chemiluminescence detection was performed using goat anti-rabbit IgG/HRP antibody (1:3000, SE134, Solarbio Science & Technology, Co., Ltd.) or goat anti-mouse IgG/HRP antibody (1:3000, SE131, Solarbio). The immunoreactive protein bands were visualized using ECL reagent (Solarbio).

### Immunofluorescent double-staining

Frozen sections of placenta tissue were washed three times with phosphate buffered saline (PBS). Slides were blocked using 5% BSA (Sangon Biotech Co., Ltd, China) for 15 min and then incubated overnight at 4°C with both LAMP3 antibody (1:100, DF7099, Affinity, China) and CK7 antibody (1:50, sc-57004, Santa Cruz, USA), respectively. Slides were then incubated with secondary antibody including goat anti-rabbit IgG H&L (FITC) (1:200, ab6717, Abcam, UK) and goat anti-mouse IgG (H+L) highly cross-adsorbed secondary antibody (1:100, A-21424, Invitrogen) at 37°C for 90 min. The nuclei were stained with DAPI, and the staining effect was viewed by a microscope (Olympus, Japan) at 400× magnification.

### MTT assay

Cell viability was monitored using MTT cell proliferation and a cytotoxicity detection kit (KeyGen Biotech, China) according to the manufacturer's instructions. The absorbance value was detected at 490 nm under a microplate reader (Biotek, USA).

### Transwell assay

The matrigel invasion assay was performed using a transwell chamber (LABSELECT, China) precoated with matrigel (Corning, USA). The cells (3×10^4^/well) were cultured into the upper well. The medium with 10% fetal bovine serum was added to the lower chamber. Incubation was performed at 37°C for 48 h, and cells were stained with crystal violet (Amresco, USA). The number of invaded cells was measured using a microscope (Olympus) at 200× magnification.

### Enzyme-linked immunosorbent assay (ELISA)

Protein levels for matrix metalloproteinase (MMP)2 and MMP9 were tested using commercial ELISA kits (MultiSciences Biotech, China) according to the manufacturer's instructions.

### TUNEL assay

TUNEL was done using the *in situ* cell death detection kit (Roche, Switzerland). Briefly, sections were incubated with TUNEL solution for 60 min, and then stained with DAPI for 5 min. The images were observed under a fluorescence microscope (Olympus) at 400× magnification.

### Statistical analysis

Data analysis was performed using GraphPad Prism 8.0 software (USA). Experiments were repeated three times and data are reported as means±SD. Unpaired *t*-test was used to compare the differences between two groups, and one-way analysis of variance (ANOVA, followed by Tukey's *post hoc* test) was used to analyze the differences among multiple groups.

## Results

### LAMP3 was upregulated in the placental tissues of patients with preeclampsia

**Figure 1:**
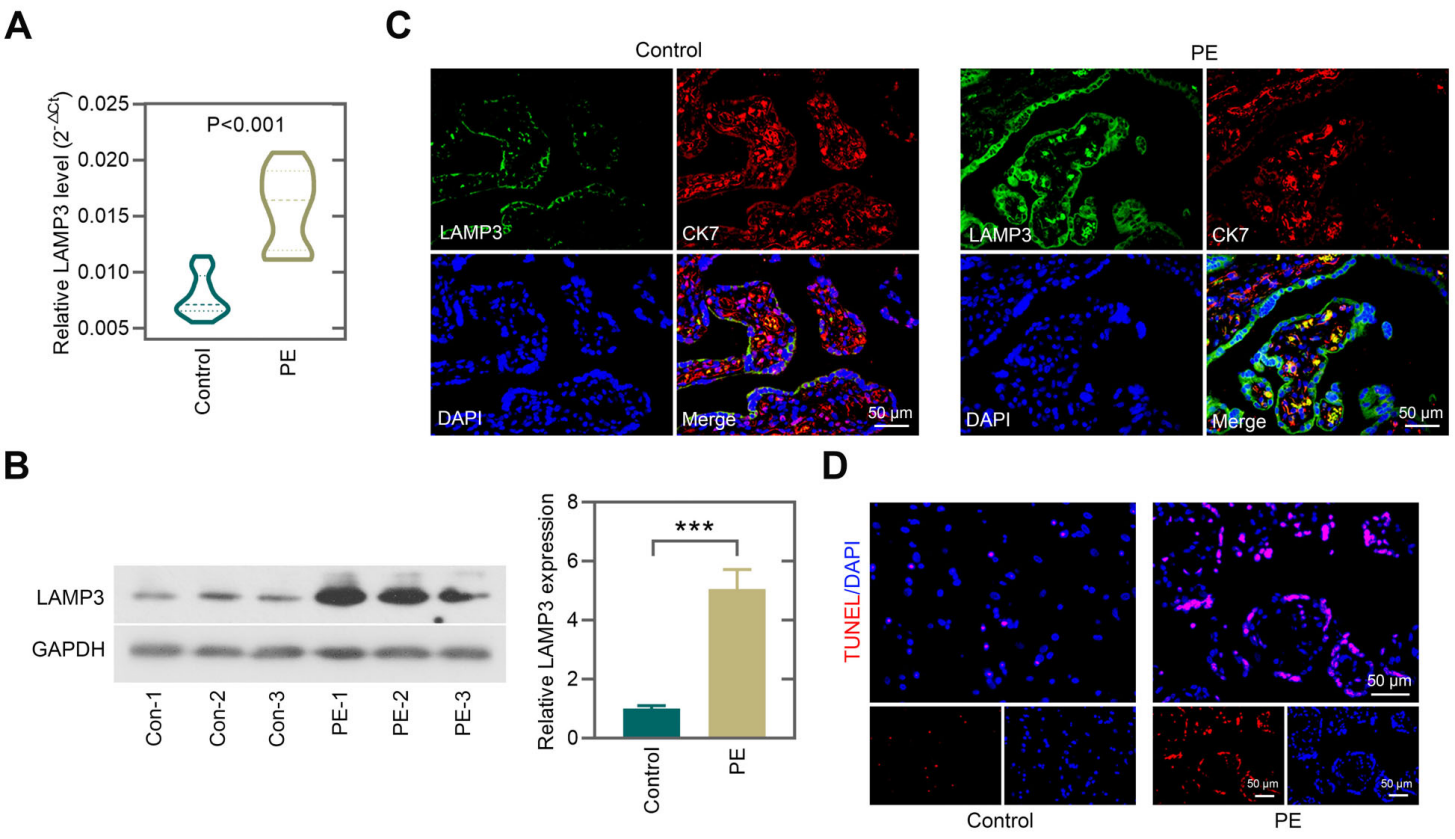
LAMP3 was upregulated in the placental tissues of patients with preeclampsia (PE). **A**, LAMP3 levels in PE placenta tissues (n=10) and normal pregnant placenta tissues (n=13). **B**, Western blot and quantitative analysis showing the upregulation of LAMP3 in preeclampsia tissues. **C**, Immunofluorescence for LAMP3 and CK7 in placenta (scale bar=50 μm). **D**, TUNEL staining was performed to detect cell apoptosis in the placenta (scale bar=50 μm). Data are reported as means±SD. ***P<0.001 (unpaired *t*-test).

The mRNA and protein levels of LAMP3 in preeclampsia placenta were upregulated compared with normal samples (Figure 1A and B). In double staining analysis, trophoblasts were identified via cytokeratin 7 (CK7) staining. Merged with the CK7 fluorescent signal, LAMP3-labeling was clearly observed. Compared with the normal placentas, LAMP3 was highly expressed in CK7-positived cells of preeclampsia placenta (Figure 1C). Preeclampsia placenta samples exhibited increased TUNEL-positive cells compared with normal controls, suggesting increased apoptosis in preeclampsia placenta (Figure 1D).

### H/R stimulation suppressed cell viability and invasion and induced apoptosis of trophoblast cells

**Figure 2:**
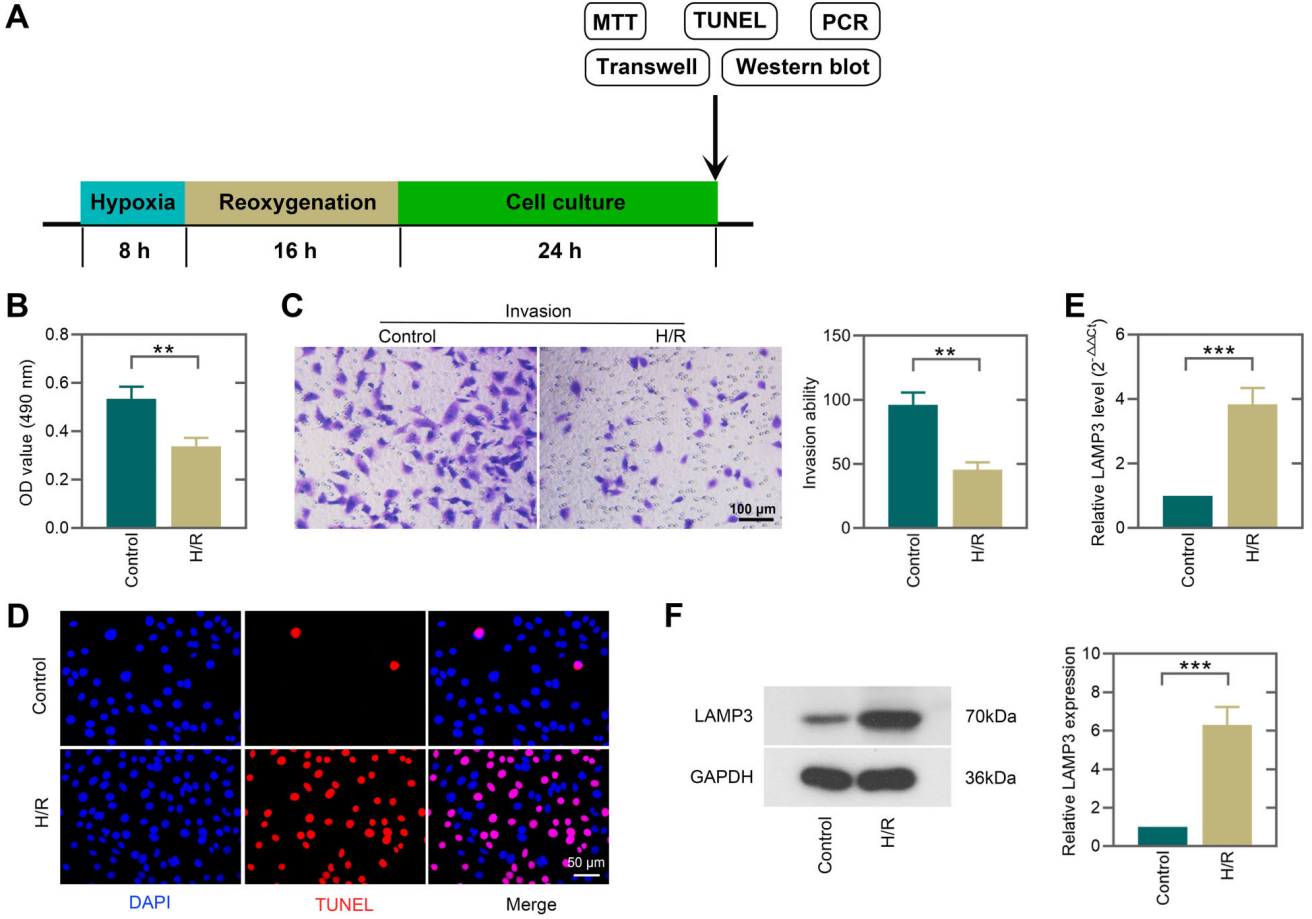
Hypoxia/reoxygenation (H/R) stimulation suppressed cell viability and invasion and induced apoptosis of trophoblast cells. **A**, Flow diagram of the establishment of H/R injured HTR-8/SVneo cell model and subsequent experiments. **B**, Cell viability of HTR-8/SVneo cells induced by H/R and subjected to MTT assay. **C**, Representative images (scale bar=100 μm) and quantitative analysis of the transwell invasion assay. **D**, Cell apoptosis was detected by TUNEL staining (scale bar=50 μm). **E** and **F**, The mRNA and protein expression levels of LAMP3 were determined by real-time PCR and western blot, respectively. Data are reported as means±SD (n=3). **P<0.01, ***P<0.001 (unpaired *t*-test).

To mimic preeclampsia *in vitro*, we established a H/R model of trophoblast cell line HTR-8/SVneo. The flow diagram is shown in Figure 2A. In the MTT assay, the cell viability was significantly suppressed after H/R stimulation (Figure 2B). Moreover, H/R treatment reduced the number of cells that invaded the lower chamber (Figure 2C). TUNEL staining indicated that cell apoptosis, as evidenced by TUNEL-positive cells, was increased under H/R condition (Figure 2D). We further examined LAMP3 expression in H/R-induced trophoblast cells. H/R exposure elevated the mRNA and protein levels of LAMP3 (Figure 2E and F). These results demonstrated that H/R exposure inhibited cell viability and invasion, promoted apoptosis of trophoblast cells, and enhanced the expression of LAMP3.

### Knockdown of LAMP3 promoted cell viability and invasion and suppressed apoptosis of trophoblast cells under H/R condition

**Figure 3:**
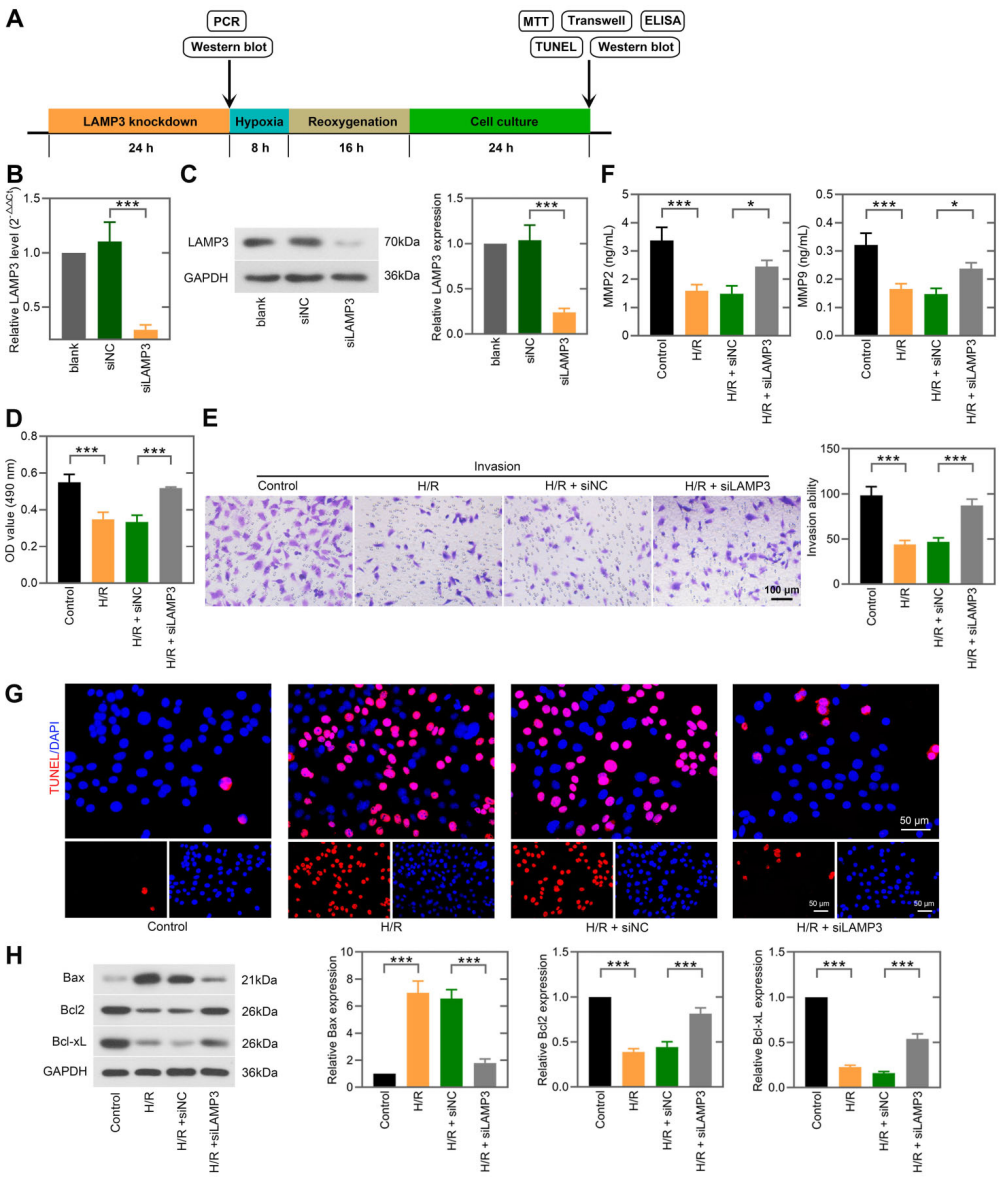
Knockdown of LAMP3 promoted cell viability and invasion and suppressed apoptosis of trophoblast cells under hypoxia/reoxygenation (H/R) condition. **A**, Flow diagram of the experiments to investigate the role of LAMP3 in trophoblast cells under H/R. HTR-8/SVneo cells were transfected with LAMP3 siRNA or NC siRNA for 24 h, and the mRNA level (**B**) and protein expression (**C**) of LAMP3 were determined. **D**, After exposure to H/R and culture for 24 h, cell viability of LAMP3-silenced HTR-8/SVneo cells was detected by MTT assay. **E**, Transwell assay (scale bar=100 μm) was performed to evaluate the invasion ability of LAMP3-silenced HTR-8/SVneo cells under H/R stimulation. **F**, The levels of MMP2 and MMP9 in the cell supernatant were quantified by ELISA. **G**, Cell apoptosis was determined by the TUNEL assay (scale bar=50 μm). **H**, Western blot analysis was used to detect the protein levels of Bax, Bcl2, and Bcl-xL in H/R-exposed HTR-8/SVneo cells. Data are reported as means±SD (n=3). *P<0.05, ***P<0.001 (one-way ANOVA).

To explore the function of LAMP3 in cell viability, invasion, and apoptosis of trophoblast cells, we used siRNA to knockdown LAMP3 (Figure 3A-C). MTT assay indicated that LAMP3 deficiency markedly enhanced cell viability of H/R-exposed trophoblast cells (Figure 3D). Transwell assay was performed to detect cell invasion after LAMP3 silencing. The reduced invasion ability in the H/R condition was enhanced by LAMP3 siRNA (Figure 3E). Considering that MMP2 and MMP9 have been proven to play critical roles in trophoblast invasion by degrading extracellular matrix (24), we detected the levels of MMP2 and MMP9 by ELISA. H/R treatment inhibited the release of MMP2 and MMP9 of trophoblast cells, which was reversed by LAMP3 knockdown (Figure 3F). The TUNEL assay demonstrated that LAMP3 knockdown resulted in a decrease in the number of TUNEL-positive cells upon H/R stimulation (Figure 3G). H/R treatment promoted the expression of pro-apoptotic Bax and inhibited the expression of anti-apoptotic Bcl2 and Bcl-xL in HTR-8/SVneo cells, all of which were reversed by LAMP3 knockdown (Figure 3H).

### 1,25(OH)_2_D_3_ promoted cell viability and invasion, suppressed cell apoptosis, and downregulated LAMP3 expression after H/R stimulation

**Figure 4:**
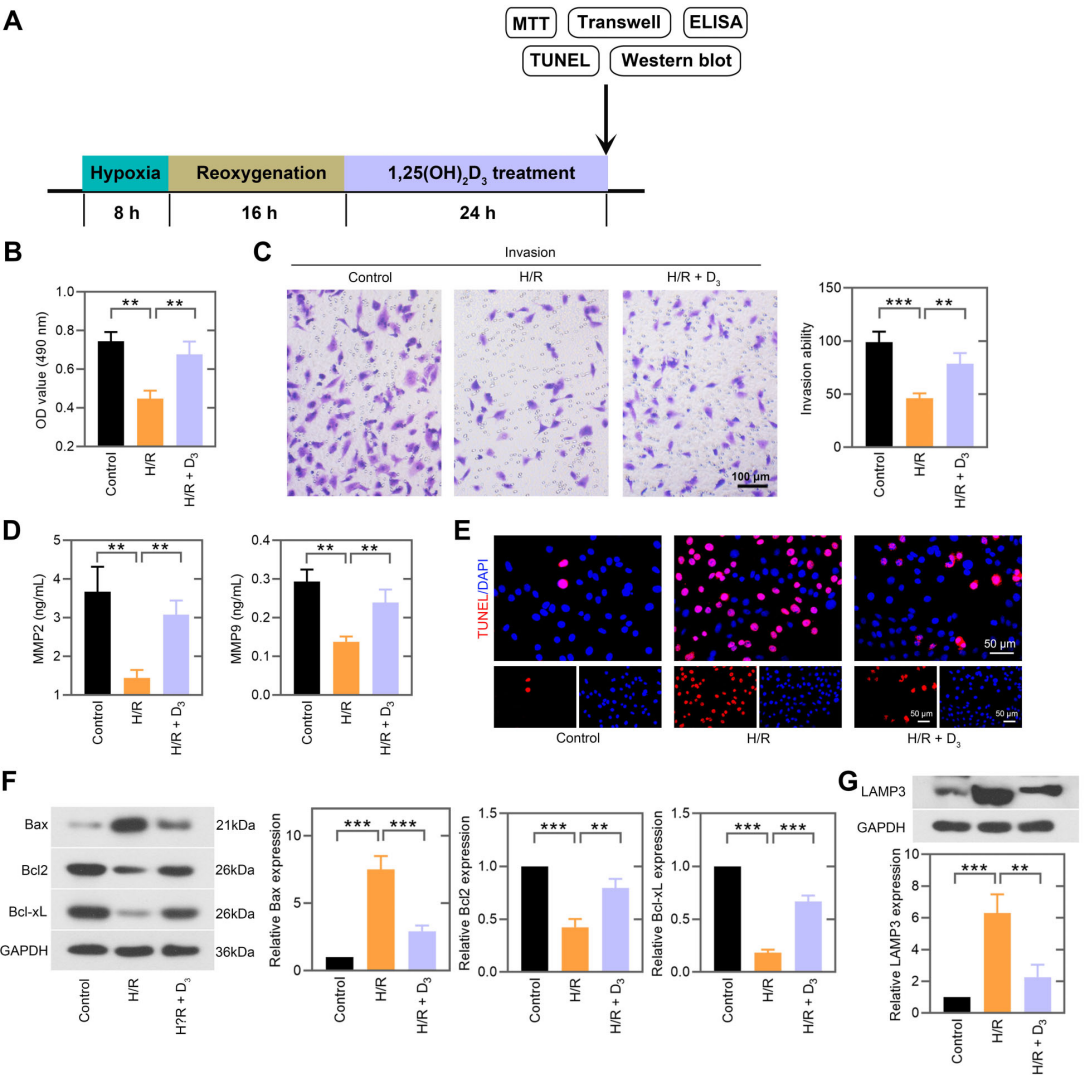
1,25(OH)_2_D_3_ promoted cell viability and invasion, suppressed cell apoptosis, and downregulated LAMP3 expression under hypoxia/reoxygenation (H/R) stimulation. **A**, HTR-8/SVneo cells under H/R condition were treated with 100 nM 1,25(OH)_2_D_3_ for 24 h and then subjected to subsequent experiments. **B**, The effect of 1,25(OH)_2_D_3_ on cell viability was detected by MTT assay. **C**, Representative images of transwell assay (scale bar=100 μm) and quantification of invasive cells. **D**, ELISA analysis was performed to detect the levels of matrix metalloproteinases (MMP)2 and MMP9. **E**, Apoptotic cells were observed by TUNEL staining (scale bar=50 μm). **F**, Western blot and quantitative analyses of Bax, Bcl2, and Bcl-xL expressions in H/R-induced HTR-8/SVneo cells with 1,25(OH)_2_D_3_ treatment. **G**, LAMP3 protein expression was examined by western blot. Data are reported as means±SD(n=3). **P<0.01, ***P<0.001 (one-way ANOVA). D_3_: 1,25(OH)_2_D_3_.

To explore whether 1,25(OH)_2_D_3_ was involved in H/R-induced trophoblast cell function, HTR-8/SVneo cells were stimulated with H/R followed by 1,25(OH)_2_D_3_ treatment (Figure 4A). MTT assay showed that the reduction of cell viability in H/R-induced cells was reversed by 1,25(OH)_2_D_3_ treatment (Figure 4B). 1,25(OH)_2_D_3_ treatment restored the inhibitory effect of H/R on cell invasion (Figure 4C), as evidenced by the increase in levels of MMP2 and MMP9 in H/R-induced cells (Figure 4D). 1,25(OH)_2_D_3_ treatment led to decreased apoptosis of H/R-exposed cells (Figure 4E), accompanied by increased expression of Bcl2 and Bcl-xL and decreased Bax expression (Figure 4F). Furthermore, we found that the protein expression of LAMP3 was downregulated by 1,25(OH)_2_D_3_ in H/R-exposed cells (Figure 4G).

### 1,25(OH)_2_D_3_ promoted cell viability and invasion and reduced apoptosis of trophoblast cells by suppressing LAMP3 under H/R condition

**Figure 5:**
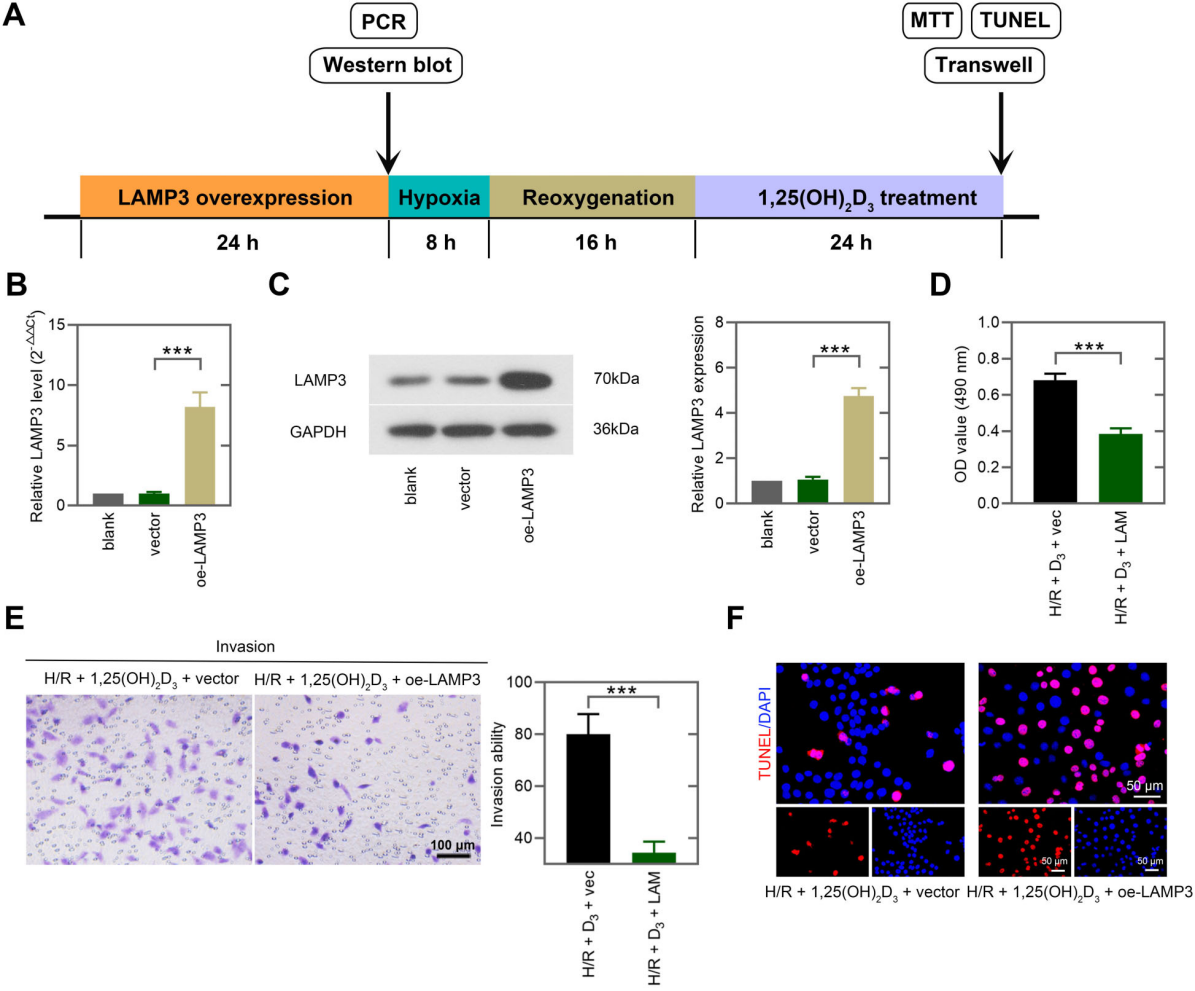
1,25(OH)_2_D_3_ promoted cell viability and invasion and reduced apoptosis of trophoblast cells by suppressing LAMP3 under hypoxia/reoxygenation (H/R) condition. **A**, Flow diagram of the *in vitro* experiments: HTR-8/SVneo cells were transfected with LAMP3 overexpressed plasmid (oe-LAMP3) or control plasmid (vector) for 24 h and exposed to H/R, followed by 1,25(OH)_2_D_3_ treatment. LAMP3 mRNA (**B**) and protein levels (**C**) were measured via real-time PCR and western blot. **D**, Cell viability was assessed by MTT assay. **E**, Transwell assay showed cell invasion ability (scale bar=100 μm). **F**, TUNEL assay was performed to assess cell apoptosis (scale bar=50 μm). Data are reported as means±SD (n=3). ***P<0.001 (one-way ANOVA and unpaired *t*-test). D_3_: 1,25(OH)_2_D_3_; vec: vector; LAM: oe-LAMP3.

To explore whether 1,25(OH)_2_D_3_ improved trophoblast cell function by downregulating LAMP3, we overexpressed LAMP3 in 1,25(OH)_2_D_3_-treated HTR-8/SVneo cells under H/R stimulation (Figure 5A). The efficacy of gene transfer was determined via real-time PCR and western blot (Figure 5B and C). The MTT assay indicated that the overexpression of LAMP3 suppressed cell viability in 1,25(OH)_2_D_3_-treated cells under H/R condition (Figure 5D). Compared with 1,25(OH)_2_D_3_-treated cells, LAMP3 overexpression abrogated 1,25(OH)_2_D_3_-mediated promotion of invasion and inhibition of apoptosis under H/R stimulation (Figure 5E and F). Therefore, we concluded that 1,25(OH)_2_D_3_ provided protective effects on the function of trophoblast cells through modulating LAMP3.

## Discussion

PE has been the focus of research due to its adverse effects on pregnant women and perinatal babies (25,26). As far as we know, this study is the first to examine the expression and role of LAMP3 in PE. Our results indicated that LAMP3 was highly expressed in the placental tissues of PE patients and further revealed its role in H/R-induced trophoblastic dysfunction. Although gene expression detection and *in vitro* results corroborated the role of LAMP3 as a pathogenic factor regulating cell viability, invasion, and apoptosis of trophoblast cells, LAMP3 is a lysosomal membrane glycoprotein with complex functions and diverse effects, therefore, *in vivo* evidence is needed to support the inference that LAMP3 contributes to disease progression in PE.

Excessive trophoblast cell apoptosis and senescence may contribute to the progression of PE (27). The increase of trophoblast apoptosis may cause both local placental injury and systemic manifestations of PE (3). Therefore, repression of trophoblast apoptosis could be a potential strategy for PE treatment. Previous studies have established the role of LAMP3 in mediating cell apoptosis (11,14,28,29). Liu et al. (14) reported that overexpression of LAMP3 inhibited apoptosis of osteosarcoma cells. Wu et al. (29) reported that the apoptosis rate was increased in LAMP3-silenced laryngeal carcinoma cells. Nakamura et al. (28) found that LAMP3 promoted apoptosis of salivary gland epithelial cells. These findings suggest that LAMP3 may have a function in promoting or inhibiting apoptosis, depending on the cell type. The role of LAMP3 in trophoblast cell apoptosis has not been explored. Our findings revealed that LAMP3 led to increased apoptosis of trophoblast cells under H/R stimulation, which is consistent with its pro-apoptotic role in salivary gland epithelial cell (28).

Accumulating studies have confirmed that deficient trophoblast cell migration and invasion may result in PE (30-32). Trophoblastic migration and invasion are often compared to tumor metastasis because they share common molecular signaling pathways and mediators (33). LAMP3 has been confirmed to regulate migration and invasion in different cancers (10,13,29). For example, deficiency of LAMP3 inhibited cell invasion in esophageal squamous cell carcinoma and laryngeal squamous cell carcinoma (10,29). Additionally, MMPs have proteolytic activity and participate in the process of trophoblast cell invasion to the uterine wall (25). The reduction of MMP-2 and MMP-9 interfered with normal spiral artery remodeling during early pregnancy, leading to preliminary pathophysiological changes in PE (34). Our study demonstrated that LAMP3 reduced the levels of MMP2 and MMP9 in H/R-exposed trophoblast cells, leading to deficient trophoblast cell invasion, which may contribute to PE development.

The exact etiopathogenesis of PE has not been fully clarified. Several promising drugs, such as vitamin D (35) and hydroxychloroquine (36), have been verified to be effective for PE prevention. The biologically active 1,25(OH)_2_D_3_ diminishes the oxidative stress-induced microparticle release from placental trophoblasts in PE (37). It also has anti-inflammatory properties in PE, which is exerted by stimulating miR-26b-5p to inhibit placental COX-2 expression (38). Our previous study revealed that 1,25(OH)_2_D_3_ effectively enhanced proliferation and vascularization abilities in H/R-treated trophoblast cells (21). Herein, we further confirmed the protective role of 1,25(OH)_2_D_3_ in trophoblast cell function, as evidenced by increased cell viability and invasion and reduced cell apoptosis under H/R exposure.

Although the molecular mechanism of vitamin D in preventing PE has not been fully established, it has been demonstrated that vitamin D has beneficial effects on PE by modulating diverse downstream effector proteins. For instance, vitamin D stimulates the expression of L-type amino acid transporter 1 (LAT1) in placental trophoblasts, thereby increasing amino acid transport across the placenta and decreasing the risk of fetal growth restriction in PE (39). Westwood et al. (40) demonstrated that vitamin D downregulates S1PR2 expression and further deranges S1P function, consequently ameliorating abnormal placentation caused by deficient migration of extravillous trophoblast. In the current study, we identified LAMP3 as a downstream effector protein of vitamin D. This is the first study to examine the effect of vitamin D on LAMP3 in trophoblast cells, which broadens our understanding of the mechanism by which vitamin D supplementation improves pregnancy outcomes. Our key finding was that LAMP3's inhibitory effects on trophoblast cell function was completely abolished by treatment of cells with 1,25(OH)_2_D_3_ and that this was mediated by downregulation of LAMP3 expression. Nevertheless, how vitamin D regulates LAMP3 expression in trophoblast cells and whether this was achieved through vitamin D receptor-mediated transcriptional regulation after binding to vitamin D or through direct binding of vitamin D to LAMP3 protein remains unknown. These questions warrant detailed exploration in future study.

In summary, our study clarified the important role of LAMP3 in the dysfunction of trophoblast cells. In particular, we provided evidence that vitamin D supplementation during pregnancy may be beneficial for decreasing trophoblast cell dysfunction by downregulating LAMP3 expression. These findings may provide a theoretical basis for the use of vitamin D in clinical prevention and treatment of PE and propose a novel therapeutic strategy by targeting LAMP3 in PE treatment.
